# Improving clerkship preparedness: a hospital medicine elective for pre-clerkship students

**DOI:** 10.1080/10872981.2017.1307082

**Published:** 2017-04-11

**Authors:** Denise M. Connor, Paul J. Conlon, Bridget C. O’Brien, Calvin L. Chou

**Affiliations:** ^a^ Division of Hospital Medicine, San Francisco VA Medical Center, San Francisco, CA, USA; ^b^ Department of Medicine, University of California San Francisco, San Francisco, CA, USA; ^c^ School of Medicine, Medical College of Wisconsin, Milwaukee, WI, USA

**Keywords:** Early clinical experience, curriculum/program evaluation, academic hospital medicine, medical education - clinical skills training, medical education - undergraduate

## Abstract

**Background**: Medical students often struggle to apply their nascent clinical skills in clerkships. While transitional clerkships can orient students to new roles and logistics, students may benefit from developing clinical skills in inpatient environments earlier in their curriculum to improve readiness for clerkships.

**Intervention**: Our four- to six-session elective provides pre-clerkship students with individualized learning in the inpatient setting with the aim of improving clerkship preparedness. Students work one-on-one with faculty who facilitate individualized learning through mentoring, deliberate practice, and directed feedback.

Second-year medical students are placed on an attending-only, traditionally ‘non-teaching’ service in the hospital medicine division of a Veterans Affairs (VA) hospital for half-day sessions. Most students self-select into the elective following a class-wide advertisement. The elective also accepts students who are referred for remediation of their clinical skills.

**Outcome**: In the elective’s first two years, 25 students participated and 47 students were waitlisted. We compared participant and waitlisted (non-participant) students’ self-efficacy in several clinical and professional domains during their first clerkship. Elective participants reported significantly higher clerkship preparedness compared to non-participants in the areas of physical exam, oral presentation, and formulation of assessments and plans.

**Conclusions**: Students found the one-on-one feedback and personalized attention from attending physicians to be a particularly useful aspect of the course. This frequently cited benefit points to students’ perceived needs and the value they place on individualized feedback. Our innovation harnesses an untapped resource – the hospital medicine ‘non-teaching’ service – and serves as an attainable option for schools interested in enhancing early clinical skill-building for all students, including those recommended for remediation.

**Abbreviations**: A&P: Assessment and plan; H&P: History and physical; ILP: Individual learning plan

## Background

Despite calls to reform pre-clerkship medical education [1], many students have limited opportunities for deliberate practice of their clinical skills in settings that mirror future clerkship environments. Transitional courses help students gain familiarity with clerkship expectations and settings [2] but generally focus on orienting students to workplace logistics and roles rather than on enhancing clinical skills. Consequently, when students arrive on inpatient services, they often have difficulty adapting their nascent clinical skills to deliver hypothesis-driven presentations that communicate their findings and clinical reasoning. Students devote significant mental energy and time to acquiring these foundational skills at the start of the clerkships, which limits their ability to fully engage with other valuable aspects of workplace learning [3–7].

We designed an elective for pre-clerkship second-year medical students based on best practices for early clinical experiences. These include: individualized learning, integration of classroom knowledge with clinical experience, situated learning in an authentic clinical setting, and hands-on opportunities to improve bedside skills through deliberate practice with intensive faculty feedback, rather than merely through observation [1,8–11]. Given the importance of an ‘invitational’ atmosphere to foster effective learning in workplace settings, we also paid careful attention to creating a welcoming environment that encouraged students’ questions and clearly identified clinical learning opportunities [11,12]. Further, rather than discouraging participants from revealing their weaknesses, we required students to identify clinical skills in need of improvement and to focus their elective experience on practicing and seeking directed feedback on those skills. This curricular orientation toward mastery and continuous self-improvement fosters a mindset that optimizes learning in clinical settings [11].

## Intervention

In our elective, pre-clerkship second-year medical students work one-on-one with faculty on an attending-only, ‘non-teaching’ hospitalist service (a clinical service dedicated to caring solely for hospitalized patients) at an academic VA medical center for half-day sessions. The elective is available for credit (pass-fail grading only) and is publicized through class emails. Most students self-select into the elective based on personal interest. Our school’s foundational clinical skills course refers a smaller subset of students for remediation of clinical skills.

Enrollment is first-come-first-served, except for referred students, who receive priority enrollment. All students share their learning goals and, when relevant, reasons for referral, with elective faculty as part of their Individualized Learning Plans (ILPs), described below.

We used an evidence-based approach for effective early authentic clinical experiences grounded in a sociocultural framework. This framework focuses on enhancing students’ sense of legitimacy, establishing clear roles, identifying discomfort in transitions between the lay and medical worlds, and consciously assessing risk and offering responsibility in a graded fashion [9]. Because individual students show better acceptability and easier integration into workplace settings than student pairs or groups, we chose to have students work one-on-one with elective faculty and patients [9]. We expected this design to support students’ sense of legitimacy in the workplace. Because pre-clerkship students typically feel constrained by the student role [9], leading them to perform incomplete history and physicals (H&Ps) for fear of inconveniencing patients, we set clear expectations for students to perform full-length H&Ps. We empowered patients to notify students if they needed a break, and reassured students that they could practice complete H&Ps unless interrupted by the patient.

Further, we encouraged students to set specific and aspirational objectives using SMART (specific, measurable, achievable, relevant, timebound) learning goals linked with prior experiences in their ILPs. We asked students to consider the elective as an opportunity to practice unfamiliar skills with the aim of self-improvement, rather than as an experience requiring them to impress faculty members with fully competent performance [11]. We also encouraged students to connect clinical findings with their pathophysiology knowledge, to communicate clinical reasoning during oral presentations, and to link classroom knowledge with clinical experience. Finally, we provided students with real-time feedback and opportunities for deliberate practice with a series of faculty attendings on the hospitalist service [13].

Before their first session, students created a pre-elective ILP that resembled future clerkship-based templates (Appendix 1) and guided them to identify their strengths, growth areas, and goals [13,14]. Remediation students also created learning plans targeted to their needs, drawing on a learner-centered approach to remediation [15]. Faculty reviewed each student’s ILP during the first session and, if needed, suggested adjustments to align goals with opportunities available during the elective.

The focus of activities varied, based on ILP objectives. For example, students who identified physical examination as a growth area may have examined a series of patients with faculty observation, followed by feedback on technique and bedside manner. Students who chose to focus on synthesizing data and communicating their reasoning conducted entire H&Ps (often with observation during history-taking), completed write-ups, and delivered oral presentations to faculty for focused feedback. This model fostered deliberate practice – providing students with opportunities for repeated practice of clinical skills that they identified in their ILPs as key goal areas, with dedicated faculty feedback on these domains across multiple sessions.

Because of the challenges of coordinating schedules between students and faculty, students often worked with different faculty members from session to session. To address this discontinuity, faculty wrote comments on students’ strengths and challenges at the end of each elective session and suggested activities for future sessions using an educational sign-out tool (Appendix 2). This process set the next session’s agenda, provided continuity of learning, and allowed students to progress with appropriate levels of challenge despite faculty changes [16].

At the end of the elective, students revisited their pre-elective ILPs, reflected on progress, and identified growth areas. They sent an updated ILP to the elective director, who offered a feedback session to review selected comments from the educational sign-out tool and consolidate plans for future improvement.

## Methods

Our study received exempt status from the University of California, San Francisco and San Francisco VA Medical Center Institutional Review Boards. The intervention group included all 25 students who participated in the first two years of the elective. Of the 25 participants, six students enrolled for remedial purposes. We used waitlisted students as a comparison group for self-assessed clerkship preparedness. Of the 47 students who were waitlisted for the elective due to space constraints, 22 consented to complete a Clerkship Preparedness Survey. We offered $10 gift cards to both elective participants and waitlisted students as an incentive for completion of the Clerkship Preparedness Survey.

We used several methods to measure the impact of our elective, and evaluated the elective at the first three levels of the Kirkpatrick framework: reaction/satisfaction, perceived learning, and perceived impact [17], using an end-of-the-elective survey, qualitative analysis of students’ ILPs, and a Clerkship Preparedness Survey given to both participating and waitlisted students.

To evaluate student satisfaction with and reaction to the course and course faculty, as well as perceived learning, we distributed anonymous, end-of-the-elective surveys to all students after their final elective session. 20/25 (80%) students completed this anonymous evaluation at the end of the elective, rating satisfaction with items on a five-point Likert scale. Our satisfaction survey was adapted from a similar instrument used in our institution for rating educational seminars. We sought feedback on the survey from several medical students and faculty members and incorporated their input into the survey questions and format prior to using it in the elective.

Because objectives for the course were individualized using student-generated ILPs, evaluating the success of the course required us to understand students’ self-identified learning goals. We therefore categorized students’ learning goals, set forth in their ILPs, using qualitative content analysis [18]. Two authors (DMC and PJC) developed categories based on the goals listed in students’ pre-elective ILPs and coded the goals into categories. The authors reconciled discrepancies in coding through discussion.

To assess perceived impact, we sent a Clerkship Preparedness Survey to participating and waitlisted (non-participating) students after the first week of their first clerkships. This survey assessed students’ self-perceptions of their clinical skills at the beginning of their first clerkship, and included questions focused on skill areas identified as learning goals in students’ ILPs. Though we did not formally validate our Clerkship Preparedness Survey, we developed the survey in consultation with medical education scholars at our institution, piloted it with several medical students and faculty members, and adapted it based on their feedback prior to using it with elective students. We compared students’ survey responses (intervention and comparison group) with an independent samples t-test, and calculated a Bonferroni correction (p ≤ 0.007) to account for multiple comparisons.

## Results

Students spent four to six half-days over a range of 2–12 weeks in the elective (determined by students’ availability; mean sessions: 4.4, range: 4–6).

In the anonymous evaluation at the end of the elective, students rated the overall quality of the elective, the usefulness of feedback provided during the elective, quality of faculty teaching, the ILP, and elective activities highly (4–5 out of 5, mean 4.25 or higher). At the learning level, students strongly agreed that they would change clinical practice due to the elective (mean = 4.75; SD: 0.55). The vast majority of students’ open-ended responses to the ‘most valuable aspect of the course’ highlighted individualized feedback from faculty. Some students also mentioned skill building in areas that mirrored goals set in pre-elective ILPs (e.g., oral presentation, history taking, assessment and plan (A&P), and physical exam).

Pre-elective ILP goals emphasized receiving individualized feedback on clinical skills from attending physicians (mentioned by 20/25 of students) and building skills in history-taking (19/25), oral presentation (17/25), physical exam (17/25), A&P (15/25), note writing (14/25), interpersonal skills and communication (7/25), medical knowledge (7/25), and use of the electronic medical record (3/25).

The Clerkship Preparedness Survey was completed by 20/25 (80%) enrolled and 22/47 (47%) waitlisted students. All students completed the survey during their first clerkship, with the majority of completions within the first three weeks of the clerkship. Students were distributed across the full range of clerkships including medicine, neurology/psychiatry (a combined clerkship), pediatrics, obstetrics/gynecology, and surgery. Elective students rated themselves significantly higher in physical exam, oral presentation, and A&P skills; most items had large effect sizes (Table 1).

**Table 1.** Results of the clerkship preparedness survey.

		N	Mean	Std. deviation	p-Value*	Effect size**
History taking *(I know how to gather useful clinical information from a new patient in a reasonable period of time)*	Participants	20	4.2	0.5	.012	.905
	Waitlisted	22	3.7	0.6		
Physical exam *(I am able to detect most major abnormalities that my residents/attendings find)*	Participants	20	3.7	0.7	.004	.992
	Waitlisted	22	2.9	0.9		
Oral case presentation *(I know what information to include when presenting a new patient to my attending)*	Participant	20	3.8	0.8	.006	.936
	Waitlisted	22	2.9	1.1		
Assessment/plan *(I know how to formulate a problem-based assessment/plan for a hospitalized patient)*	Participant	20	3.5	0.8	.001	1.104
	Waitlisted	22	2.5	1.0		
Stress/Well-being *(I feel overwhelmed and/or very anxious about how to succeed in clerkships)*	Participant	20	2.8	1.1	.052	.636
	Waitlisted	22	3.5	1.1		
Confidence in my abilities *(While I still have a lot to learn, I feel confident that my abilities in patient care are at the right level for my stage in training)*	Participant	20	3.8	0.8	.036	.705
	Waitlisted	22	3.2	0.9		
***Composite: Professionalism items (combines six items, described in legend)	Participant	20	4.0	0.4	.028	.702
	Waitlisted	22	3.6	0.7		

Respondents rated agreement with each item on a five-point Likert scale from Strongly disagree [1] to Strongly agree [5].

*p-Value:  With Bonferroni correction for multiple comparisons, significance is reached at 0.05/7 = 0.007.

**Effect size: Given similar standard deviations between our groups, effect size measured using Cohen’s d.

***‘Professionalism’ combined responses to six items on self-efficacy/attitudes about self-assessment, seeking out feedback, creating an individualized learning plan (ILP), and creating strategies for meeting learning goals.

Students also responded to two open-ended questions in the Clerkship Preparedness Survey about how the elective prepared them for clerkships and the most useful aspects of the elective. Students most commonly valued learning how to construct A&P’s, practicing oral presentations and history-taking, and receiving individualized feedback from an invested attending physician (Table 2).

**Table 2.** Thematic analysis with representative quotes to open-ended questions in the Clerkship Preparedness Survey.

Theme	Representative Quotations
Learning how to construct an Assessment and Plan (A&P)	*The practice of developing an A&P was valuable as it is not a skill we practice often when presenting during the first two years*. *The elective was the first time I had done a formal assessment and plan, and I’m glad I was able to practice a few times before third year started.*
Practice with oral presentations	*Simply forcing the student to sit down and write out a note and orally presenting it was enormously helpful in exposing weak points. It wasn’t until I was expected to come up with a full note and then present it formally that I realized how unwieldy and foreign many aspects of either task were for me*. *Practicing…and then receiving extensive feedback about all aspects of [the oral presentation] was incredibly valuable immediately before heading out onto the wards.*
Individualized attention and feedback from an invested attending	*Provided individualized attention from attendings and longitudinal assessment of goals identified in our ILP*. *Attendings were great about being familiar with my specific goals and discussing progress, as well as remaining weaknesses*. *One-on-one teaching with faculty and dedicated time [for] feedback*
Practice with history-taking	*Practice in taking a full history from a new patient*. *History taking with pertinent positives and negatives*. *Being able to interview…real patients*

Representative elective student responses to: (1) In what ways did the elective course ‘Hospital-Based Medicine: A Clinical Skills Tutorial’ help to prepare you for clerkships? and (2) What was the most useful aspect of the course ‘Hospital-Based Medicine: A Clinical Skills Tutorial?’ grouped by the most commonly mentioned themes.

## Discussion

Students rated our inpatient elective highly and reported higher self-efficacy in physical exam, oral presentation and A&P at the start of clerkships compared to non-participating students. Compelling evidence that students achieved their aims for the elective includes alignment of perceived benefits with goals stated in ILPs, written comments about the most valuable aspects of the elective, and survey results demonstrating that participants felt significantly more prepared than their non-participating peers. Since no referred students were waitlisted, the enrollee pool was enriched with remediation students. Even with the additional training provided by the elective, some remediation students may still have felt less prepared for clerkships than their colleagues, so this difference between our two groups may have led us to underestimate the elective’s impact.

Our elective deepens pre-clerkship learning by providing students with real-time, specific feedback from faculty that is based on direct observation of the skills students choose as focus areas. The elective offers opportunities for clinical skill development for high-achieving students as well as those needing remediation. Our innovation builds on the preceptorship model, which similarly provides one-on-one mentoring and focuses on clinical skill development [1,19,20], but with the important difference that outpatient-based preceptorships focus on applying clinical skills in a different context with an emphasis on chronic care.

Some schools have created longitudinal clinical coaching programs which pair students with faculty mentors from the beginning of medical school to support clinical skill development [21], which reduces the need for pre-clerkship electives like ours. However, in the absence of an early clinical coaching program, our elective provides an impactful early opportunity for clinical skill building and optimizes academic hospitalists’ opportunities for teaching. While longitudinal experiences with a single hospitalist coach would be preferable [16], we utilized an educational sign-out tool to improve the continuity of students’ learning (Appendix 2). While we did not study faculty impressions of the elective, several faculty anecdotally noted that involvement while on a ‘non-teaching’ service was a salve against burnout.

Because ‘low-stakes’ pass/fail experiences may reduce anxiety and maximize learning, we opted to structure our innovation as an elective [2]. However, while this elective structure can provide learner benefits, it is important to consider whether certain students are systematically excluded from elective experiences, and whether providing supplemental opportunities to motivated students who seek out extra practice risks opening an achievement gap. We reduced this unintended consequence by reserving slots for remediation students. However, it would be helpful to survey students who did not enroll to determine if there were barriers to enrollment, and to track how they performed in clerkships.

Our study has several limitations. Our elective took place in a single institution (an academic VA medical center affiliated with a single medical school) on one faculty-led inpatient medicine service, limiting our innovation’s generalizability. Institutions wishing to develop a similar experience may need to modify the elective to fit their specific clinical context. In addition, we report on a relatively small sample size of elective students, introducing potential selection bias. Further, we chose students who expressed interest in the elective but were waitlisted due to lack of space as a comparison group. It is possible that these students differed from participants, though the timing of accepted versus waitlisted students’ emails (email timestamps determined first-come-first-served enrollment for non-remediation students) were clustered over a short period, suggesting similar interest in enrollment. Our data represent students’ self-assessments and self-efficacy, rather than objective evidence of skill development. Evaluation of enrolled students’ skills by clerkship faculty would add weight to our analysis. Finally, given the anonymous nature of our surveys, we did not separately analyze remediation students’ responses. Future study to determine if remediation students’ performance improved is warranted.

## Conclusions

The elective’s popularity, evidenced by our waitlist, speaks to pre-clerkship students’ thirst for authentic clinical skill-building. The value of connecting students with invested attendings for direct observation, feedback, and teaching in the inpatient setting shines through as a key lesson for medical school faculty who focus on early learners. Situating learning coupled with deliberate practice in the inpatient setting is a rich opportunity not only to develop and potentially remediate clinical skills, but also to encourage the natural motivation of learners to engage with patients and clinical faculty.

## Prior presentations

An abstract describing the model for this educational innovation was presented at the 2013 University of California, San Francisco Academy of Medical Educators Educational Showcase.

## Appendices

### Appendix 1. Student Individualized Learning Plan (ILP) and Critical Reflection Tool

***Acknowledgement*** *: Adapted from University of California, San Francisco (UCSF), School of Medicine ILP Tool for Clerkships; originally developed by Patricia A. Robertson, Laura B. Cantino, H. Carrie Chen, Vanja C. Douglas, Robert Daroff, and Karen Hauer, and first presented at the UCSF, Academy of Medical Educators’ annual symposium in 2012*

**Instructions**:

Please answer the following questions. Email your completed ILP to the course director before your first session. These questions are designed to help you to identify, reflect upon, and develop a plan to address your learning needs. Use as much space as you need.

You will use your ILP as a starting point to help you and the faculty focus your elective experience. We strongly recommend that you upload your ILP into your portfolio. At the end of the elective, you will re-visit your ILP, set new goals for the future, and discuss those goals with course faculty.

Based on your clinical experiences so far (i.e. Standardized and Real Patient Interviews, OSCE, Preceptorship), what do you think are your areas of strength in the clinical arena?

Consider which competencies (below) present the greatest challenges for you and describe specific challenges for you in *one or two* of the competency domains.

**Table:**

Competency domains	Challenge
Patient care	
Medical knowledge	
Practice-based learning & improvement	
Interpersonal & communication skills	
Professionalism	
Systems-based practice	

Based on these broad challenges, choose two to three skills you would like to work on during this elective. For each skill, list one to two ways you will work to improve your skills. Be as specific as possible.

1.

2.

3.

**End of the Elective Reflection**

**Instructions**:

Review your initial ILP goals. Assess the progress you have made toward your learning goals. (Include as much objective evidence as you can – i.e. you can include a progress note you have written, feedback you’ve received.)

1.

2.

3.

List three take-home points that you will carry with you from your experience in this elective. *(We will send you reminder emails over the coming year to remind you of these take-home points and check in on your progress!)*

1.

2.

3.

Which clinical learning goals will you continue to work on, or what new learning goals would you now like to shift your focus toward as you move toward entering clerkships? Include a plan for how you will achieve each of these three goals:

1.

2.

3.

### Appendix 2. Educational sign-out tool

**Confidential: Hospital-based medicine: A clinical skills tutorial:**

**Student assessment & educational sign-out**

**Student name:**

**Comments on SP-Encounter Video** (if available)

**Focus areas (Based on Saxena et al., *Remediation techniques for student performance problems after a comprehensive clinical skills assessment*, Acad Med. 2009 May; 84**[5]**:669–76**):

__ History-taking

__ Physical exam

__ Clinical knowledge

__ Clinical reasoning (A/P)

__ Professionalism

__ Communication

Notes on why these focus areas were selected:

Based on ILP goals:

Educational plan for targeting these areas:

**Learning Log:**

(What did the student spend time on during this session (i.e. conducted two new H&Ps, saw follow-up patient, presented, and completed a SOAP note?)

Date: Worked with: Activity:

Date: Worked with: Activity:

Date: Worked with: Activity:

Date: Worked with: Activity:

**End of Elective: Reflection on progress made in target areas**:

Do the initial target areas seem correct (or have you found that the student struggles much more in a focus area other than the one initially selected?)
